# Development and Validation of LC-Q-TOF-MS Methodology to Determine Mycotoxin Biomarkers in Human Urine

**DOI:** 10.3390/toxins14100651

**Published:** 2022-09-20

**Authors:** Nuria Dasí-Navarro, Manuel Lozano, Sabrina Llop, Ana Esplugues, Alessandra Cimbalo, Guillermina Font, Lara Manyes, Jordi Mañes, Pilar Vila-Donat

**Affiliations:** 1 Laboratory of Food Chemistry and Toxicology, Faculty of Pharmacy, University of Valencia, 46100 Burjassot, Spain; 2 Epidemiology and Environmental Health Joint Research Unit, FISABIO—Universitat Jaume I—Universitat de València, 46020 València, Spain; 3 Spanish Consortium for Research on Epidemiology and Public Health (CIBERESP), 28029 Madrid, Spain

**Keywords:** women urine, mycotoxins, biomonitoring, simple extraction, untargeted

## Abstract

Mycotoxin contamination of foodstuffs is a health concern worldwide and monitoring human exposure to mycotoxins is a key concern. Most mycotoxins and their metabolites are excreted in urine, but a reliable detection method is required, considering the low levels present in this biological sample. The aim of this work is to validate a sensitive methodology capable of simultaneously determining ten targeted mycotoxins as well as detecting untargeted ones by using Liquid Chromatography coupled to Quadrupole Time of Flight Mass Spectrometry (LC-Q-TOF-MS). The targeted mycotoxins were: enniatin A, B, A1, and B1, beauvericine, aflatoxin B1, B2, G1 and G2, and ochratoxin A. Several extraction procedures such as liquid-liquid extraction, dilute and shoot, and QuEChERS were assessed. Finally, a modified simple QuEChERS extraction method was selected. Creatinine adjustment and matrix-matched calibration curves are required. The limit of detection and limit of quantification values ranged from 0.1 to 1.5 and from 0.3 to 5 ng/mL, respectively. Recoveries achieved were higher than 65% for all mycotoxins. Later, the method was applied to 100 samples of women’s urine to confirm the applicability and determine their internal exposure. The untargeted mycotoxins most found were trichothecenes, zearalenones, and ochratoxins.

## 1. Introduction

Mycotoxin exposure from food occurs globally and produces adverse effects on human and animal health. Besides the regulated mycotoxins, several other non-regulated mycotoxins reported as emerging ones deserve special attention for their effects. Food and food products are contaminated by simultaneous mycotoxins due to the ability of fungus to produce different mycotoxins. Food can be infected by various fungi simultaneously or at various stages, with the mycotoxins remaining stable in the food during crop growth, post-harvest, and packing [1]. Furthermore, processing does not eliminate their presence in food, as observed in ready-to-eat food samples in which aflatoxins (AFs), ochratoxin A (OTA), deoxynivalenol (DON), 3-acetyl-deoxynivalenol (3ADON), 15-acetyl-deoxynivalenol (15ADON), T-2 and HT-2 toxins, neosolaniol (NEO), diacetoxyscirpenol (DAS), nivalenol (NIV), enniatins (ENNs), beauvericin (BEA), zearalenones (ZEAs), and *Alternaria* toxins were detected in cereals, legumes, dry fruits, fresh fruits, vegetables, wine, beer, fish, and meat [2,3,4].

Well-known legislated OTA and AFs are classified by the International Agency for Research on Cancer (IARC) as possibly carcinogenic to humans (group 2B) and carcinogenic to humans (Group 1), respectively [5] and are part of the group of regulated mycotoxins with maximum concentration values for a wide number of foods [6]. Aflatoxin B1 (AFB1) and OTA toxicity and their potentially harmful effects have been shown in in vivo and in vitro models, being AFB1 the most toxic mycotoxin [7,8]. Another in vivo study showed that OTA had nephrotoxic effects, probably due to carcinogenic changes in the epithelial tissues [9]. Recent studies have reported that one of the main excretion routes for AFs and OTA is urine, which is shown as a suitable matrix for its determination [10,11].

In addition, special attention should be paid to non-regulated mycotoxins such as ENNs and BEA, whose presence in food products has been detected [12]. Recent studies about emerging mycotoxins have shown that enniatin B (ENNB) induced genotoxic effect in vivo after acute oral administration in mice [13], and transcriptional and protein expression changes in rats related to metabolism after in vivo assays [14].

Personal or collective total intake determination of a mycotoxin involves a long process of food sampling and analysis, with the added problem of not considering its bioaccessibility and bioavailability. The analysis of biological samples, such as blood, urine, and hair, entails a rapid approach to mycotoxin exposure in a population and allows forecasting of possible correcting measures in a specific geographical or social environment.

Human biomonitoring (HB) is a scientific technique recently applied in several recent multi-mycotoxin studies [15,16,17,18,19]. Analysis of mycotoxins in human urine is a suitable alternative to assess the exposure assessment to mycotoxins since most of them and their metabolites are efficiently excreted in this biological matrix [20]. However, a reliable detection method is required, considering the low levels present in urine [21].

Regarding extraction procedures for multi-mycotoxin analysis in human urine samples, extraction methods as solid phase extraction (SPE) [22], stable isotope dilution assay (SIDA)–SPE method [23], salting-out liquid-liquid extraction (SALLE) [24], pH-dependent liquid-liquid extraction (LLE) [25], dilute and shoot (DS) [26,27] and Quick Easy Cheap Effective Rugged and Safe (QuEChERS) have been previously employed [28,29,30,31].

For mycotoxin determination techniques in urine, liquid chromatography (LC) coupled with a fluorescence detector has been previously employed for OTA determination [32,33,34,35], as well as gas chromatography with tandem mass spectrometry for *Fusarium* mycotoxins and their metabolites [36,37]. Liquid chromatography-mass spectrometry (LC-MS/MS) is the method of choice for multi-mycotoxin determination in biological samples of major and minor mycotoxins [22,38,39].

High-Resolution Mass Spectrometry (HRMS) detectors such as Quadrupole Time-of-Flight (Q-TOF) are new techniques for multi-mycotoxin qualitative and quantitative analyses [40]. This technique presents advantages over other methods, such as the ability to record full scan spectra by measuring the accurate mass of analytes and the screening of untargeted compounds allowing a high degree of structural confirmation [41]. Notwithstanding, Q-TOF is an expensive piece of equipment requiring operators with high expertise to handle it and process the data.

The aim of this work is to validate a rapid methodology based on modified QuEChERS, able to perform qualitative and quantitative analysis suitable for HB. A method capable of determining the concentration of ten selected mycotoxins (four AFs, OTA, four ENNs, and BEA) was developed to fulfill this using LC-Q-TOF-MS. Furthermore, this technique enables us to simultaneously perform a screening of current untargeted mycotoxins and metabolites in studied samples. The method was then applied to 100 samples of urine to determine mycotoxins exposure in the female population.

## 2. Results

### 2.1. Optimization of Extraction Procedures

Different methods were tested to optimize the extraction of enniatin A (ENNA), enniatin A1 (ENNA1), ENNB, enniatin B1 (ENNB1), BEA, AFB1, aflatoxin B2 (AFB2), aflatoxin G1 (AFG1), aflatoxin G2 (AFG2), and OTA. Optimization tests were carried out through recovery assays in triplicates. For this, a blank urine sample with a creatinine concentration previously determined (0.70 mg/mL) was spiked at 25 ng/mL of mentioned mycotoxins before extraction. Then, these peak areas were compared with peak areas of urine samples spiked after extraction (matrix-matched line). Recoveries results for the different extraction methods assayed are shown in Table 1.

Dilute and shoot was tested with and without formic acid. The results obtained with water/acetonitrile/formic acid (H_2_O/ACN/HCOOH) (94:5:1, *v*/*v*/*v*) that provides excess recoveries for OTA and AFG2 can be seen in Table 1. In the absence of formic acid, the recoveries obtained for ENNA, ENNB1, and AFB1 improved slightly. However, too high recovery values were obtained for BEA, OTA, AFG1, and AFG2. Moreover, a poor chromatographic resolution was found in accordance with Greer et al. [42], as well as progressive column deterioration and signal weakening [22]. LLE provides acceptable results for ENNs, BEA, and OTA but low recovery for AFs.

Acidified QuEChERS with 0.1% of acetic acid or non-acidified presents results that could be considered globally comparable, and although the presence of acid releases better recovery values for ENNs, BEA, and OTA, a negative aspect are recovery values below 60% for AFBs.

The most acceptable average results for the set of ten mycotoxins combined were obtained with simple QuEChERS. Therefore, this was the extraction method of choice for further analysis since the purpose of this study was to develop a multi-mycotoxin method suitable for large-scale HB. For the optimization phase of the simple QuEChERS, different salt combinations as NaCl, MgSO_4_, and C18 were tested, achieving better results with 0.3 g MgSO_4_ and 0.03 g C18, in accordance with results previously obtained by Escrivá et al. [30].

### 2.2. Method Validation

The validation results obtained for the simple QuEChERS selected method are presented in Table 2. Calibration curves showed good linearity, with determination coefficient (r2) values higher than 0.998 for all mycotoxins and higher than those obtained in similar validation procedures [26,43]. The limits of detection (LODs) achieved ranged from 0.1 to 1.5 ng/mL, and the limits of quantification (LOQs) ranged from 0.3 to 5 ng/mL. These LOD/LOQ value ranges are lower than those reported by Njumbe Ediage et al. [44], indicating a good sensitivity of the method considering the low concentrations normally found in urine, and without the need to employ more extensive sample clean-up steps. Recovery results for the three concentrations tested (5, 10, 25 ng/mL) with an intra-day assay ranged from 65% to 115%. Signal suppression-enhancement (SSE, %) calculations showed signal enhancement for ENNA and signal suppression for AFB2 and AFG2. For matrix-matched calibration curves and recoveries, the same blank urine used in extraction method tests was employed to avoid signal variations due to the matrix. Matrix-matched calibration curves were employed to compensate for these effects for sample quantification purposes.

### 2.3. Optimization of LC-Q-TOF-MS Parameters

Several parameters of the chromatographic determination were tested, such as a different mobile phase consisting of H_2_O (A) and methanol (MeOH) (B) acidified with 0.1 and 0.2% formic acid alone or buffered with 5 mM ammonium formate, as well as varied gradient elution percentages, injection volumes (5, 10 and 20 μL) and flow rates of 0.2, 0.3 and 0.4 mL/min were tested. Acidification of both phases (A and B) with 0.1% HCOOH provides the best resolutions in less than 15 min. Regarding the Q-TOF-MS settings, different source and spectral parameters were tested, as detailed in Table 3, to achieve satisfactory results for all mycotoxins. Injections were performed in both positive and negative ESI mode, but the best results were obtained for all ten mycotoxins in ESI+; hence positive mode was selected to sample analysis. 

Adequate confirmation of the analytes (theoretical mass, precursor ion, retention time (RT), ion species, and polarity) is presented in Table 4. Mycotoxin profiles were evaluated through the analysis of chromatographic and spectra data (Figure S1) by injecting analytical standards to compare RT and precursor ions present in samples to those in standard solutions. 

### 2.4. Creatinine Determination

Creatinine measurement is commonly used to adjust mycotoxins concentrations due to variability in urine dilution grade [45]. Hence, creatinine levels were determined to ensure selected samples were not either too concentrated or diluted and ranged between similar concentrations to compare analytic results. However, creatinine-adjusted concentrations of mycotoxins have also been calculated, as well as creatinine excretion in 24 h considering individual body weight, since creatinine content is related to muscle mass.

Gender-independent cut-off range is often employed to discard diluted urine samples below 200 mg/L, but this bears the risk of discriminating against women since they have, in general, lower urinary creatinine concentrations compared with men. Nevertheless, creatinine concentrations of the 100 studied samples ranged from 360 to 750 mg/L (Table 5), in accordance with the results obtained by Arndt et al. [46].

Considering this, a more accurate index that considers individual body weight was employed to select 100 samples with concentrations like average creatinine values for women (10–20 mg/kg/day) [47]. Samples with values ranging from 13.9 to 18.3 mg/kg/day were selected for further analysis, as seen in Table 5.

### 2.5. Analysis of Mycotoxins in Urine Samples 

Results for incidence and quantification of ten targeted mycotoxins are shown in Table 6 (Figure S2). In 30 of the samples, at least one mycotoxin was quantified, and the co-occurrence of two or more quantified mycotoxins was shown in five samples. ENNB was quantified in 27 samples with concentrations ranging from 0.5 to 2.5 ng/mL, ENNB1 in five samples (0.5–0.8 ng/mL), AFB1 in 1 sample (0.6 ng/mL), AFB2 in 2 samples (0.3–1.4 ng/mL) and AFG1 in one sample (0.5 ng/mL). ENNA, ENNA1, BEA, OTA, and AFG2 were not found in the samples’ upper LOQ levels. 

Additionally, to obtain a more accurate index and facilitate comparisons, a mycotoxin creatinine-adjusted concentration was determined for the 30 samples containing at least one quantifiable mycotoxin, based on mycotoxin urine concentration (ng/mL) and creatinine urine concentration (mg/mL), as explained in Section 5.5, obtaining the results presented in Table 7.

As this method is suitable for biomonitoring, besides targeted analysis, a screening of some other mycotoxins present in the samples was performed, employing the pre-settled mycotoxins Metlin Personal Compound Database Library (PCDL). According to this, results for identified mycotoxins are presented in Table 8. In 79% of the samples, at least one mycotoxin or metabolite was detected, of which, 24% presented a co-occurrence of three or more mycotoxins. Achieving co-occurrence of five mycotoxins in two samples. Mycotoxins with higher incidence were deepoxy-deoxynivalenol (DOM-1) present in 46% of studied samples, ochratoxin B (OTB) (19%), and ochratoxin *α* (OT*α*) (15%). The PCDL employed for the identification also included some other micotoxins and metabolites which were not detected in any sample, such as alternariol (AOH), fumonisin B1 (FB1), fumonisin B3 (FB3), 3ADON, DON-3-glucoside, aflatoxin M1 (AFM1), aflatoxin M2 (AFM2) and aflatoxin P1 (AFP1).

## 3. Discussion

QuEChERS methodology has been widely used for the determination of pesticides and veterinary drugs in food and beverages as a previous step to the determination by LC coupled to MS with very good results against compounds of different polarity. Classic QuEChERS extraction involves two steps. The first step is an organic solvent extraction via a “salting out” using salts. The second step is a dispersive SPE clean-up to remove interferences. In the present study, we propose a simple QuEChERS extraction method, meaning only one step, using a simultaneously organic solvent, salts, and SPE phase. Simple QuEChERS is quicker and inexpensive in comparison with other extraction methods. In the LLE method, evaporation step is time-consuming, and SPE columns are inadvisable for a large number of samples owing to the cost. Reducing the extraction to a single step makes the procedure much more useful to be used in biomonitoring for risk assessment purposes, which usually requires a high number of analyses. Based on it, simple QuEChERS extraction and LC-Q-TOF-MS determination methods were successfully optimized to validate in terms of linearity, sensibility, recoveries, and SSE (%) according to in-force legislation as described above.

In previous studies concerning human urine in Spain employing dispersive liquid-liquid microextraction (DLLME) and HPLC-MS/MS, authors were able to quantify ENNB in 40% and ENNB1 in 20% of the samples, determining concentrations of 0.1–0.54 μg/L and 0.1–0.34 respectively. In comparison, ENNA1 was detected in 10% of samples at values between LOD-LOQ [30]. Another research performed by Rodríguez-Carrasco et al. [48] in urine samples from Italy using SALLE extraction and LC-Q-Orbitrap-MS/MS analysis found ENNB in 83.7% of samples with concentrations ranging from <LOQ to 0.391 ng/mL. These results agree with the present study as ENNB is the most quantifiable mycotoxin, found in 27 samples with concentrations ranging from 0.5 to 2.5 ng/mL (Table 6). ENNB was also the most identified mycotoxin (40%) in a study carried out in China by Liu et al. [49], in agreement with the present work.

Regarding AFs results, previous studies showed that despite being regulated, AFs are still found in human urine but with low incidence, as presented in this study in Table 6 (1–2%). Values are in accordance with a recent study by Al-Jaal et al. [15] analyzing different biological samples of the Qatari population employing HPLC-MS/MS, being lower than 3% for AFB1, AFB2, and AFG2 with concentrations <LOQ for AFB1 and AFB2 and 0.19–0.34 ng/mL for AFG2, finding less mycotoxin incidence in urine than in plasma. In a study of 94 samples in Portugal, AFB1 had an incidence of 12%, followed by AFB2 (3%), AFG2 (3%), and AFG1 (2%) [50], and similar results were obtained by Foerster et al. [51] who detected AFB1 in 8% of samples. Moreover, as seen in the previously cited literature, in this study, AFs have also been quantified in low concentrations (0.5–1.4 ng/mL), which is to be expected since these are legislated mycotoxins.

Contrary to developed countries in which mycotoxins are more controlled, in sub-Saharan Africa, the lack of adequate mycotoxin regulations causes higher diet exposure [52]. In a study carried out in mother-infant pairs in Nigeria, regulated mycotoxins as AFs, DON, and OTA were frequently detected, observing a concentration range of maternal urinary DON of 75–608.85 ng/L, being 2–10 times higher than previously observed in other studies in Africa [16]. Similar results were obtained in a biomonitoring study in Nigerian children, where a higher incidence of aflatoxin Q1 (AFQ1) (68%) in infant urine compared with aflatoxin M1 (AFM1) (9%) suggests aflatoxin exposure in young children may be underreported [53].

Regarding creatinine-adjusted concentrations of the quantifiable samples, ENNB and ENNB1 had mean concentrations of 3.5 ng/mg and 1.3 ng/mg, respectively; as for AFB1, AFB2 and AFG1, concentrations were 1.1, 1.6, and 1.1 ng/mg respectively (Table 7). Lower results were obtained in an exposure assessment performed in Chile, with an AFB1 mean concentration of 0.3 ng/mg [51]. Regardless of the use of creatinine-adjusted results for other mycotoxins, this approach has been also employed to determine *Alternaria* mycotoxins in the Chinese population [17] and for OTA and citrinin (CIT) in Bangladesh [54].

Regarding the results of this study presented in Table 8, DON and DOM-1 overall (52%) represented the higher incidence of detected mycotoxins. Indeed, DON and its metabolite DOM-1 have been previously studied to assess exposure in the Chinese population based on diet and urinary biomarkers, with an incidence of 95% for DON and 14.6% for DOM-1 [55]. DOM-1 was also the most detected mycotoxin in an exposure study carried out in Spain, being detected in 53% of samples, as reported by Carballo et al. [56]. Regarding other DON metabolites, untargeted screening detected a 15ADON incidence of 4%. However, it did not find 3ADON or DON-3-glucoside. Related to DON glucuronide metabolites, a pilot survey was carried out in Croatia, in which mycotoxin biomarkers were analyzed in urine samples from pregnant women, finding DON-15-glucuronide and DON-3-glucuronide were detected in 97.5% of the studied samples, revealing high DON exposure in this cohort [57].

In relation to OTA, as presented in Table 8, only its metabolites, OTB and OT*α*, were detected in the samples, but with an incidence of 34% altogether, as for ZEA and its metabolite *α*-ZOL, which combined showed up in 11% of samples (Table 8). Similar results were found by Šarkanj et al. [57] in the Croatian pregnant women study mentioned above, who only detected OTA in 10% of samples at trace levels, confirming co-exposure of OTA and DON in those samples. Besides, in a biomonitoring study performed in Sweden, where DON was found to be the most frequently detected mycotoxin (63%), followed by OTA (51%), ZEA (37%), *α*-ZOL (21%), and DOM-1 (8%), and showing co-exposure of mycotoxins [58].

The results obtained in the present study and the data found in the bibliography confirm the presence of mycotoxins in human urine in countries with different crops and climates, which shows that the usual intake of contaminated foods by toxigenic fungi occurs all over the world. Nevertheless, more studies are needed to correlate biomarkers in urine with the bioavailability of mycotoxins, bearing in mind that urine is a useful fluid for eliminating less fat-soluble mycotoxins and their hydro-soluble phase I and II derivatives from the body.

## 4. Conclusions

A methodology based on simple QuEChERS extraction followed by LC-Q-TOF-MS to determine ENNA, ENNA1, ENNB, ENNB1, BEA, AFB1, AFB2, AFG1, AFG2, and OTA in human urine, was used with good recovery results. Furthermore, with its application to 100 urine samples and creatinine adjustment, it was possible to quantify at least one mycotoxin in 30% of samples, being ENNs the most occurrent mycotoxins, followed by AFs in 4% of samples. All this shows the importance of the control carried out on the mycotoxins legislated in food products, and that to reduce the level of food contamination, it could be of interest to introduce new mycotoxins in the state regulations.

The quick multi-mycotoxin method developed is suitable for at least 11 untargeted mycotoxins, DOM-1 being the compound mostly found (46%), followed by OTB (19%) and OTα (15%).

## 5. Materials and Methods

### 5.1. Chemicals and Reagents

ACN and MeOH, both LC/MS grade, were supplied by Fisher Scientific (Loughborough, UK). Deionized water was obtained at the laboratory using a Milli-QSP^®®^ Reagent Water System (Millipore, Bedford, MA, USA). Formic acid (CH_2_O_2_, grade > 98%) was obtained from Acros organics (Geel, Belgium). Ethyl acetate (C_4_H_8_0_2_) (HPLC grade > 99%) from Alfa Aesar (Karlsruhe, Germany). Magnesium sulfate, anhydrous (>99.5%) (MgSO_4_), was obtained from Thermo Fisher (Kandel, Germany). The C18-E (50 µm, 65 Å) was purchased from Phenomenex (Torrance, CA, USA). Ammonium formate crystalline (HCO_2_NH_4_) (>99%) was supplied by Alfa Aesar (Karlsruhe, Germany). Acetic acid glacial (CH_3_COOH, grade > 99%) was supplied by Fisher Scientific (Loughborough, UK). Nylon syringe filters 13 mm 0.22 µm from Membrane Solutions (Valencia, Spain). Creatinine 98% from Acros organics (Loughborough, UK), Picric acid (C_6_H_2_OH(NO_2_)_3_) (98%) from Panreac (Barcelona, Spain), and NaOH from VWR (Radnor, PA, USA).

### 5.2. Standards and Solutions

Analytical standards of ENNA, ENNA1, ENNB, ENNB1, BEA, AFB1, AFB2, AFG1, AFG2, and OTA were obtained from Sigma Aldrich (St. Louis, MO, USA). A combined standard working solution was prepared in MeOH at 100 µg/mL, and serial dilutions of this mix were prepared. All working solutions were protected from light and stored at −20 °C. 

### 5.3. Sampling

Urine samples (n = 100) were obtained from female adult participants in the INMA (INfancia y Medio Ambiente-Environment and Childhood) project (http://www.proyectoinma.org/, accessed on 20 September 2022) a network of seven birth cohorts in Spain that aims to study the role of exposure to environmental pollutants in the air, water, and diet during pregnancy and childhood for child and adolescent growth and development [59]. Urine samples were collected from subjects in Valencia County (mean age 35.6 ± 4 years), with body weight ranging from 50 to 93 kg (mean 64.5 ± 12 kg). Samples were stored at −20 °C until analysis, and those with similar creatinine values were the ones selected for this analysis. All urine samples were centrifuged at 10,640× *g* for 10 min at 4 °C, and the supernatant was collected to proceed with the creatinine determination and the extraction method of choice. Informed consent was obtained from all participants in each phase, and the study was approved by the hospital ethics committees in the participating regions.

### 5.4. Sample Extraction Essays

For method validation, the same blank urine sample, obtained from a 30-year-old volunteer woman from Spain, who avoided cereal-based product intake, including wine, beer, and coffee, four days before urine sample collection, was employed throughout all tests. The lack of mycotoxins was verified (Figure S3), as well as creatinine concentration determination to know the dilution grade of the urine. Urine was centrifuged at 10,640× *g* for 10 min at 4 °C, and the supernatant was collected to proceed with the different extractions.

#### 5.4.1. Simple QuEChERS

Firstly, 1 mL of previously centrifuged urine was added to a tube with 0.3 g of MgSO_4_ and 0.03 g of C18 and vortexed, then 1 mL of ACN was added and then vortexed for 1 min. Afterward, samples were centrifuged at 3200× *g* for 5 min at 4 °C. The upper layer containing the organic phase was separated and filtered with a nylon syringe filter of 13 mm 0.22 µm to an HPLC vial. 

#### 5.4.2. Acidified QuEChERS

For acidified QuEChERS extraction, the procedure was performed the same way as for simple QuEChERS mentioned before but employing ACN acidified with acetic acid at different concentrations of 0.2, 0.1, 0.05%.

#### 5.4.3. Liquid-Liquid Extraction

Firstly, 1 mL of previously centrifuged urine at 10,640× *g* for 10 min at 4 °C was added in a tube with 0.5 g of NaCl and vortexed, then 2.5 mL of ethyl acetate was added and then vortexed and agitated for 20 min on a shaking incubator (ArgoLab, Arezzo, Italy). At last, the sample was centrifuged at 3200× *g* for 10 min at 4 °C, and the organic phase was filtered and evaporated under an N_2_ stream (99.9% purity) and reconstituted with 200 µL of MeOH/H_2_O (50:50, *v*/*v*). 

#### 5.4.4. Dilute and Shoot

Dilute and shoot was carried out through centrifuging the urine at 10,640× *g* for 10 min at 4 °C. Two different solvent proportions were tested according to what proposed in the literature [24], H_2_O/ACN/HCOOH (94:5:1, *v*/*v*) and H_2_O/ACN (90:10, *v*/*v*). For this method, 100 µL of previously centrifuged urine were mixed with 900 µL of solvent in each case. 

### 5.5. Creatinine Determination

In this study, creatinine determination of urine samples was carried out as follows: previously centrifuged urine (10,640× *g* for 10 min at 4 °C) was diluted (1:5), and 500 µL were mixed with 1250 µL of milli-Q water, with the addition of 250 µL of alkaline picrate (0.20 g picric acid mixed with 250 mL NaOH 1N). A calibration curve was made to quantify the samples using increasing concentrations of a 50 µg/mL creatinine pattern mixed with milli-Q water and 250 µL of alkaline picrate to prepare the following concentrations: 3.12, 6.25, 12.50, 18.75, 25.00, 31.25 µg/mL. Creatinine determination is based in the formation of a yellow-orange complex with creatinine and picric acid and whose absorbance is measured at 500 nm wavelength. For measurement, a VWR UV-1600PC spectrophotometer was employed in this work. Once creatinine of the 100 samples was determined, a creatinine-adjusted concentration (ng/mg) was calculated to obtain a more accurate index and facilitate comparison with the literature based on mycotoxin concentration of positive samples (ng/mL) and creatinine concentration (mg/mL) [60].

### 5.6. Method Validation

The simple QuEChERS method was selected for sample extraction. Method validation was carried out, characterized in terms of linearity, precision, sensibility through LOD and LOQ, recoveries, and SSE (%) according to the Commission Decision 2002/657/EC [61]. LOD and LOQ were determined using matrix-matched calibration curves, and those were calculated defining a minimum signal/noise (S/N) of three and ten, respectively. 

For all mycotoxins, matrix-matched calibration curves (blank urine spiked after sample extraction) and standard curves (MeOH:H_2_O, 50:50 *v*/*v*) were made in a linear range of 0.1–50 ng/mL for ENNs and AFs and 1.2–50 ng/mL for BEA and OTA.

Extraction recoveries (blank sample spiked before extraction) were carried out at concentrations of 5, 10, and 25 ng/mL in triplicates (intra-day analysis). SSE (%) parameter was calculated to evaluate the possible matrix effect that enhances or suppresses the analytic signal, calculated by comparing the calibration curves slopes as follows:

(1) $$\mathrm{SSE}\;\left(\%\right)=\frac{\mathrm{slope}\;\mathrm{of}\;\mathrm{matrix}\;\mathrm{matched}\;\mathrm{calibration}\;\mathrm{curve}}{\mathrm{slope}\;\mathrm{of}\;\mathrm{MeOH}:\mathrm{water}\;\mathrm{calibration}\;\mathrm{curve}\;}\cdot 100$$

### 5.7. Separation and Detection Parameters for LC-Q-TOF-MS 

The chromatographic determination was carried out on an Agilent 1200 LC system (Agilent Technologies, Santa Clara CA, USA) coupled to a 6540 Ultra High Definition Accurate-Mass Q-TOF-MS and set up with the Dual Agilent Jet Stream Electrospray Ionization (Dual AJS ESI) source interface. 

The LC system was equipped with a degasser, binary pump, and autosampler. The column used was a Phenomenex (Torrance CA, USA) Gemini^®®^ NX-C18 (3 μm, 30 × 2 mm ID) with a guard column Security Guard™ Ultra C18. Mobile phases were water (A) and ACN (B), both with 0.1% of formic acid. The injection volume was 10 μL and flow was 0.3 mL/min. The gradient started at 0 in phase B and increased linearly to 3% in 3 min; after, phase B increased to 50% in 4 min; then, phase B increased to 75% in 5 min. After holding for 3 min, it returned to the initial parameters in 3 min. The total run time was 18 min.

The MS detection was carried out in positive ionization mode with the following settings: gas drying temperature was 325 °C; drying gas flow (N_2_) was 5 L/min; nebulizer pressure was 60 psi; sheath gas temperature was 300 °C; sheath gas flow was 12 L/min. Ion source parameters were as follows: capillary voltage, 2500 V; nozzle voltage, 1200 V; fragmentor voltage, 170 V; skimmer voltage, 70 V and Oct1RFVpp, 750 V. Collision energy voltage for fragmentation was 10 V. For spectral parameters, the acquisition was carried out in the mass range 100–900 m/z at a scan rate/time of five spectra/s. An internal mass correction was performed with reference masses 121.0509 and 922.0098 m/z.

Besides biological samples, matrix-matched and standard calibration curves were injected as routine during sample analysis to ensure the correct performance of the instrument. 

### 5.8. Targeted Mycotoxins Quantification

For data processing, firstly, a custom database was created, employing the Agilent PCDL Manager with Metlin mass spectral library data. In the edited PCDL for this study, the ten validated mycotoxins were included along with other mycotoxins for untargeted analysis. To obtain metabolomics identification by comparing molecular mass spectra of the samples with those in the Metlin database, as well as manual integration of peak chromatograms for authentication, Agilent Mass Hunter Workstation Software was used. Thus, validated mycotoxins (ENNA, ENNA1, ENNB, ENNB1, BEA, AFB1, AFB2, AFG1, AFG2, and OTA) present in the samples were verified by comparing RT, mass, and m/z with values obtained by standards injection and biochemical databases. Afterward, proper quantification was performed through matrix-matched calibration curves for each targeted mycotoxin, making it possible to quantify those mycotoxins with results above LOQ.

### 5.9. Screening for Untargeted Identification 

Besides targeted identification, in order to determine the occurrence of other mycotoxins present in the samples, a screening study was performed, employing the custom PCDL database, which besides the ten targeted mycotoxins, included some other mycotoxins (DAS, HT-2, ALT, NEO, ZEA, DON, AOH, FB1, and FB3) and metabolites such as DOM-1, 15-ADON, 3ADON, DON-3-glucoside, OTB, OTα, α-ZOL, AFM1, AFM2, and AFP1. Agilent Mass Hunter Workstation Software allows an untargeted scan of unknown analytes to be ran by performing a full spectra screening and then identifying compounds with the pre-settled Metlin PCDL [50]. Mycotoxins and metabolites results provided by the software, with scores higher than 70%, were qualified as identified compounds.

## Figures and Tables

**Table 1:** Recoveries (%) comparison results for the different extraction methods tested.

Mycotoxins	Recoveries at 25 ng/mL (%)			
	Simple QuEChERS	Acidified QuEChERS	LLE	Acidified Dilute and Shoot
ENNA	70.4 ± 2.2	80.2 ± 3.2	75.2 ± 1.8	64.8 ± 0.9
ENNA1	77.6 ± 4.3	85.8 ± 6.2	88.9 ± 0.9	88.2 ± 0.1
ENNB	74.6 ± 2.4	86.3 ± 2.6	85.5 ± 1.5	104.2 ± 3.1
ENNB1	80.8 ± 3.5	87.4 ± 1.9	87.8 ± 0.5	100.5 ± 3.4
BEA	105.6 ± 4.4	93.5 ± 3.0	84.1 ± 0.5	126.4 ± 6.1
OTA	75.7 ± 5.3	87.9 ± 5.9	91.6 ± 2.9	298.0 ± 9.9
AFB1	65.4 ± 3.5	46.2 ± 0.5	26.2 ± 2.0	54.7 ± 12.1
AFB2	79.5 ± 3.3	56.9 ± 0.5	40.4 ± 1.2	88.2 ± 15.2
AFG1	79.9 ± 0.9	45.3 ± 1.3	31.2 ± 0.2	125.2 ± 18.2
AFG2	88.1 ± 1.3	47.7 ± 1.8	32.9 ± 1.2	217.9 ± 17.5

LLE: liquid-liquid extraction.

**Table 2:** Method validation results.

Mycotoxin	Calibration Curve	Linearity (r^2^)	LOD (ng/mL)	LOQ (ng/mL)	Recovery (%)			SSE (%)
					5 (ng/mL)	10 (ng/mL)	25 (ng/mL)	
ENNA	y = 13661x − 2024.1	0.999	0.1	0.3	70.9 ± 4.4	72.0 ± 0.2	70.4 ± 2.2	162
ENNB	y = 13947 x − 6233.2	0.999	0.1	0.3	81.1 ± 6.4	80.5 ± 2.0	74.6 ± 2.4	124
ENNA1	y = 11171x − 4586.9	0.999	0.1	0.3	77.5 ± 4.6	77.7 ± 1.9	77.6 ± 4.3	117
ENNB1	y = 19186x − 8300.7	0.999	0.1	0.3	82.9 ± 3.5	82.7 ± 0.1	80.8 ± 3.5	123
BEA	y = 8518.2x − 26562	0.999	1.2	3.8	87.9 ± 7.7	91.7 ± 0.3	105.6 ± 4.4	121
OTA	y = 135.56x − 1751.4	0.998	1.5	5.0	115.3 ± 5.5	86.8 ± 7.7	75.7 ± 5.3	82
AFB1	y = 1580x − 509.11	0.999	0.1	0.3	85.8 ± 0.6	84.8 ± 1.9	65.4 ± 3.5	97
AFB2	y = 906.1x + 799.27	0.999	0.1	0.3	79.1 ± 1.1	77.4 ± 3.5	79.5 ± 3.3	67
AFG1	y = 1530.5x − 538.23	0.999	0.1	0.3	75.6 ± 1.6	79.4 ± 0.4	79.9 ± 0.9	96
AFG2	y = 787.92x + 710.55	0.998	0.1	0.3	80.1 ± 7.3	90.9 ± 4.2	88.1 ± 1.3	63

LOD, limit of detection. LOQ, limit of quantification. SSE, signal suppression-enhancement.

**Table 3:** Optimization of LC-Q-TOF-MS parameters.

Parameters	Tested Parameters	Selected Parameters
Gas temperature (°C)	330; 325	325
Drying gas (L/min)	5; 10	5
Nebulizer (psi)	30; 60	60
Sheath gas temperature (°C)	295; 300; 350	300
Sheath gas flow (L/min)	9;12	12
Capillary voltage (V)	2500; 3500	2500
Nozzle voltage (V)	500;1200	1200
Fragmentor voltage (V)	160; 170	170
Skimmer voltage (V)	30; 70	70
Mass range (m/z)	50–1000; 100–900	100–900
Collision energy (V)	5; 10	10

**Table 4:** LC-Q-TOF-MS mycotoxins parameters ESI positive mode.

Mycotoxin	Formula	Theoretical Mass	Precursor Ion (m/z)	Retention Time (min)	Ion Species
ENNA	C_36_H_63_N_3_O_9_	681.4548	682.46 699.49	12.1	[M + H] + [M + NH4]+
ENNB	C_33_H_57_N_3_O_9_	639.4082	640.41 657.44	11.4	[M + H] + [M + NH4]+
ENNA1	C_35_H_61_N_3_O_9_	667.4388	668.44 685.47	11.9	[M + H] + [M + NH4]+
ENNB1	C_34_H_59_N_3_O_9_	653.4235	654.43 671.45	11.7	[M + H] + [M + NH4]+
AFB1	C_17_H_12_O_6_	312.0628	313.07	1.3	[M + H]+
AFB2	C_17_H_14_O_6_	314.0769	315.08	1.2	[M + H]+
AFG1	C_17_H_12_O_7_	328.0562	329.06	1.1	[M + H]+
AFG2	C_17_H_14_O_7_	330.0714	331.08	1.0	[M + H]+
BEA	C_45_H_57_N_3_O_9_	783.4073	784.41 801.44	11.8	[M + H] + [M + NH_4_]+
OTA	C_20_H_18_ClNO_6_	403.0826	404.08	9.1	[M + H]+

**Table 5:** Creatinine of urine samples (n = 100).

Concentration Range (mg/L)	Mean Concentration (mg/mL)	Mean Individual Body Weight (kg)	Excretion Range (mg/kg/day)	Mean Excretion (mg/kg/day)
360–750	0.5 ± 0.1	64.5 ± 11.9	13.9–18.3	16.0 ± 1.5

**Table 6:** Incidence and concentration (ng/mL) of targeted mycotoxins in studied samples (n = 100).

Mycotoxins	No. Samples	Concentration Range (ng/mL)
ENNB	27	0.5–2.5
ENNB1	5	0.5–0.8
AFB1	1	0.6
AFB2	2	0.3–1.4
AFG1	1	0.5

**Table 7:** Urinary mycotoxins after creatinine-adjusted concentrations.

Mycotoxins	Corrected Concentration (ng/mg)
ENNB	3.5 ± 1.0
ENNB1	1.3 ± 0.4
AFB1	1.1
AFB2	1.6 ± 1.3
AFG1	1.1

**Table 8:** Data base screening of untargeted mycotoxins in studied samples (n = 100).

Detected Mycotoxins	Incidence (nº Samples)
15ADON	4
DAS	1
HT-2	3
ALT	2
NEO	9
DON	6
DOM-1	46
OTB	19
OT*α*	15
ZEA	3
*α*-ZOL	8

15ADON: 15-acetyl-deoxynivalenol, DAS: diacetoxyscirpenol, ALT: altenuene, NEO: neosolaniol, DON: deoxynivalenol, DOM-1: deepoxy-deoxynivalenol, OTB: ochratoxin B, OT*α*: ochratoxin *α*, ZEA: zearalenone, *α*-ZOL: *α* zearalenol.

## Supplementary Materials

The following are available online at https://www.mdpi.com/article/10.3390/toxins14100651/s1, Figure S1: HPLC-Q-TOF-MS chromatogram of 10 validated mycotoxins (Enniatin A, Enniatin B, Enniatin A1, Enniatin B1, Beauvericine, Aflatoxin B1, Aflatoxin B2, Aflatoxin G1, Aflatoxin G2 and Ochratoxin A) in a spiked urine sample at 50 ng/mL; Figure S2: HPLC-Q-TOF-MS chromatogram of 3 positive samples containing Aflatoxin B1 (0.6 ng/mL), Aflatoxin B2 (1.4 ng/mL) and Aflatoxin G1 (0.5 ng/mL); Figure S3: HPLC-Q-TOF-MS chromatogram of blank urine sample.
