# Supplementary material for: Development and Validation of LC-Q-TOF-MS Methodology to Determine Mycotoxin Biomarkers in Human Urine
Source: Toxins (Basel). 2022 Sep 20;14(10):651. doi: 10.3390/toxins14100651 (PMC9612178; doi:10.3390/toxins14100651)
Supplement: Supplementary file 1 [file toxins-14-00651-s001.zip › toxins-1903411-supplementary.pdf]

Supplementary Materials: Development and validation of LC-Q-TOF-MS methodology to determine mycotoxin biomarkers in human urine

Nuria Dasí-Navarro, Manuel Lozano, Sabrina Llop, Ana Esplugues and Alessandra Cimbalo

Guillermina Font, Lara Manyes, Jordi Mañes,* and Pilar Vila-Donat

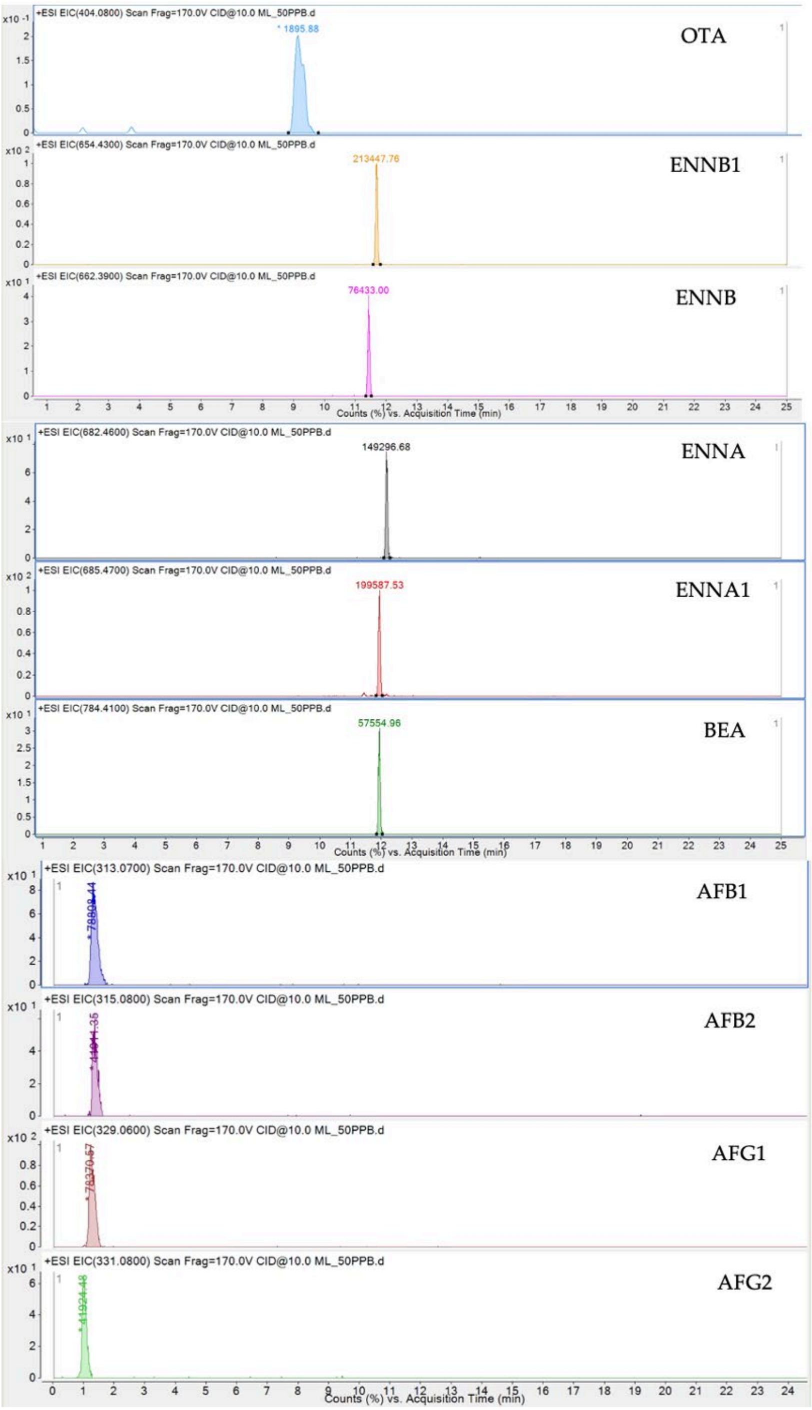

Figure S1. HPLC-Q-TOF-MS chromatogram of 10 validated mycotoxins (Enniatin A, Enniatin B, Enniatin A1, Enniatin B1, Beauvericine, Aflatoxin B1, Aflatoxin B2, Aflatoxin G1, Aflatoxin G2 and Ochratoxin A) in a spiked urine sample at 50 ng/mL.

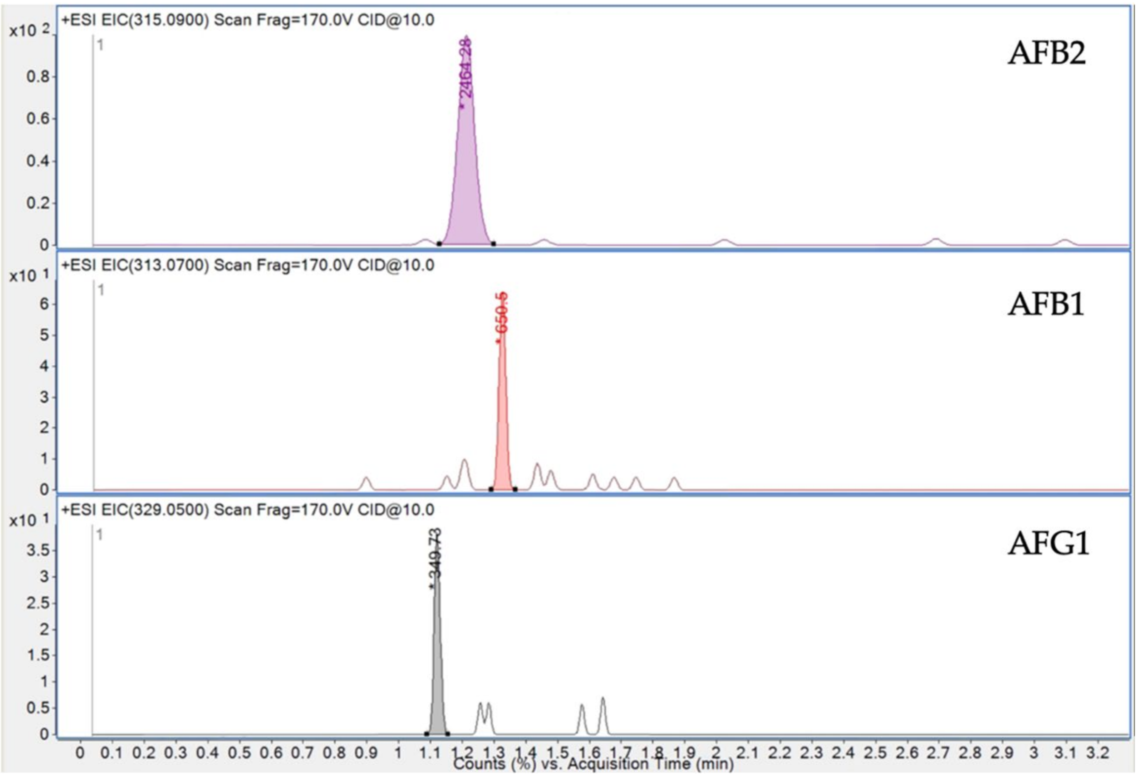

Figure S2. HPLC-Q-TOF-MS chromatogram of 3 positive samples containing Aflatoxin B1 (0.6 ng/mL), Aflatoxin B2 (1.4 ng/mL) and Aflatoxin G1 (0.5 ng/mL).

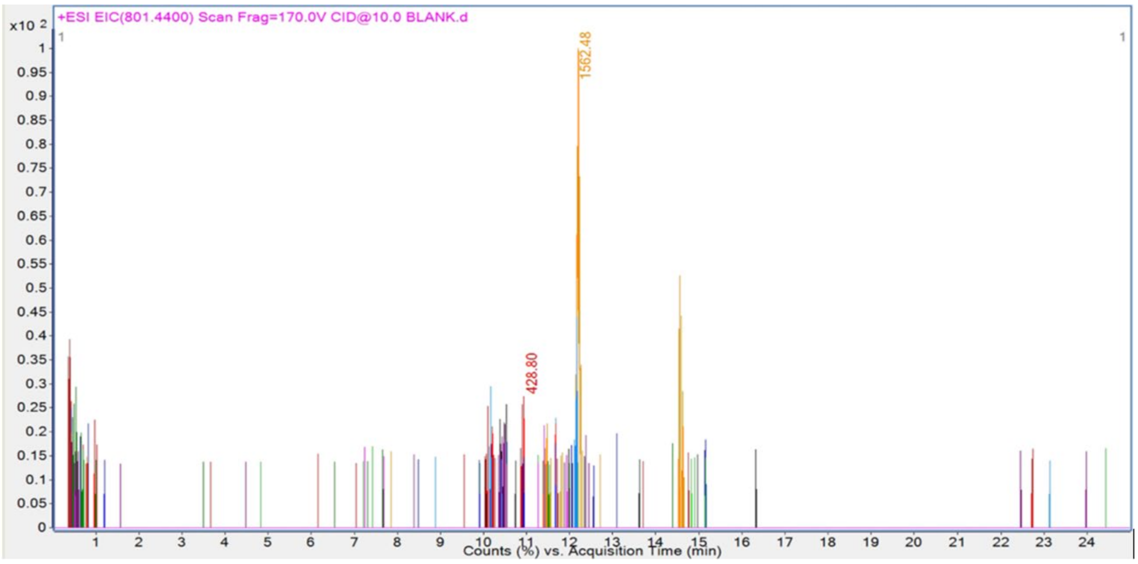

Figure S3. HPLC-Q-TOF-MS chromatogram of blank urine sample.
